# Hyperglycemic chorea

**DOI:** 10.1093/omcr/omab118

**Published:** 2021-12-11

**Authors:** Tokio Sasaki, Yuji Suzuki, Mitsunobu Sato

**Affiliations:** 1 Department of Internal Medicine, Iwate Prefectural Kuji Hospital, Kuji, Iwate, Japan; 2 Division of Hepatology, Department of Internal Medicine, Iwate Medical University School of Medicine, Iwate, Japan; 3 Division of Neurology and Gerontology, Department of Internal Medicine, Iwate Medical University School of Medicine, Yahaba, Iwate, Japan

**Figure 1:**
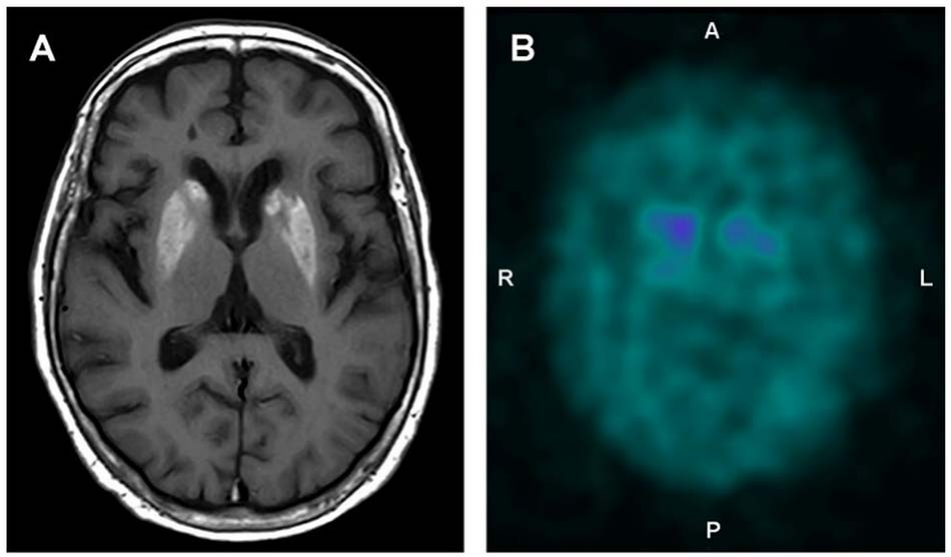
Magnetic resonance images of the brain of a 78-year-old woman with hyperglycemic chorea revealing (A) hyperintensity on T1-weighted image in the basal ganglia bilaterally. (B) Dopamine transporter single-photon emission computed tomography showing marked reduction of striatal accumulation bilaterally.

A 78-year-old woman with type 2 diabetes and poor compliance with the prescribed diet presented at a primary care clinic with a complaint of anorexia. She appeared disoriented; however, her physical examination was unremarkable. Laboratory tests revealed a hyperosmolar hyperglycemic state with plasma glucose at 44 mmol/L, calculated plasma osmolarity of 332 mOsm/L, and absence of ketoacidosis. Her serum glycated hemoglobin was 16%. Three days following the initiation of hypoglycemic treatment with insulin, she observed uncontrollable movements of her arms. Neurological examination revealed involuntary, bilateral, choreic movements affecting her upper extremities (Supplementary Video 1). The movements worsened with activity; however, they did not occur during sleep. Her mental status, verbal response, and other physical parameters were normal. Magnetic resonance imaging (MRI) revealed a hyperintense T1 signal in the basal ganglia bilaterally (Fig. 1A). These findings were consistent with hyperglycemic chorea. Choreic movements persisted even after hyperglycemia correction and treatment with haloperidol. Dopamine transporter single-photon emission computed tomography (DAT-SPECT) revealed marked reduction of striatal accumulation bilaterally (Fig. 1B), suggesting presynaptic dopaminergic dysfunction involvement in the pathogenesis of hyperglycemic chorea. Accordingly, dopamine replacement was considered as a therapeutic option. After initiating levodopa therapy, the choreic movements disappeared.

Hyperglycemic chorea is a rare complication of diabetes mellitus, characterized by acute onset chorea and striatal hyperintensity on T1-weighted MRI [1]. One of the putative mechanisms of hyperglycemic chorea is striatal neuron dysfunction [2]. Blood glucose level control is the principal treatment of hyperglycemic chorea. Additional anti-chorea medications, such as haloperidol, are considered for refractory cases [3]. The present patient had haloperidol-resistant hyperglycemic chorea and was treated with levodopa based on DAT-SPECT information. Hyperglycemic chorea with reduced striatal accumulation in DAT-SPECT has been reported [4]. Our results suggest that Dopamine replacement therapy based on a DAT-SPECT result is a feasible treatment option for hyperglycemic chorea.
